# Elamipretide reduces pyroptosis and improves functional recovery after spinal cord injury

**DOI:** 10.1111/cns.14221

**Published:** 2023-04-20

**Authors:** Wu Jiang, Fan He, Guoming Ding, Junsong Wu

**Affiliations:** ^1^ Department of Orthopedics, The First Affiliated Hospital Zhejiang University School of Medicine Hangzhou China; ^2^ Department of Orthopedics, Affiliated Hangzhou First People's Hospital Zhejiang University School of Medicine Hangzhou China

**Keywords:** elamipretide, mitochondrial dysfunction, neuroinflammation, pyroptosis, spinal cord injury

## Abstract

**Aims:**

Elamipretide (EPT), a novel mitochondria‐targeted peptide, has been shown to be protective in a range of diseases. However, the effect of EPT in spinal cord injury (SCI) has yet to be elucidated. We aimed to investigate whether EPT would inhibit pyroptosis and protect against SCI.

**Methods:**

After establishing the SCI model, we determined the biochemical and morphological changes associated with pyroptosis, including neuronal cell death, proinflammatory cytokine expression, and signal pathway levels. Furthermore, mitochondrial function was assessed with flow cytometry, quantitative real‐time polymerase chain reaction, and western blot.

**Results:**

Here, we demonstrate that EPT improved locomotor functional recovery following SCI as well as reduced neuronal loss. Moreover, EPT inhibited nucleotide‐binding oligomerization domain‐like receptor 3 (NLRP3) inflammasome activation and pyroptosis occurrence and decreased pro‐inflammatory cytokines levels following SCI. Furthermore, EPT alleviated mitochondrial dysfunction and reduced mitochondrial reactive oxygen species level.

**Conclusion:**

EPT treatment may protect against SCI via inhibition of pyroptosis.

## Graphical abstract

Elamipretide (EPT) reduced neuronal loss and improved locomotor functional recovery following SCI. Moreover, EPT inhibited nucleotide‐binding oligomerization domain‐like receptor 3 (NLRP3) inflammasome activation and pyroptosis occurrence and decreased pro‐inflammatory cytokines levels following SCI. Furthermore, EPT alleviated mitochondrial dysfunction and reduced mitochondrial reactive oxygen species level. Together, our data indicated that EPT may provide a novel approach to promote functional recovery after SCI.

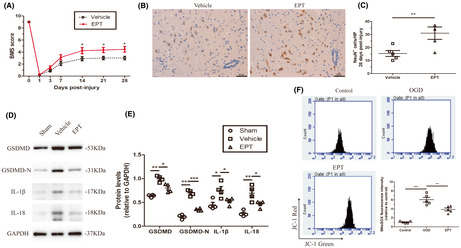

## 1 INTRODUCTION

Spinal cord injury (SCI) is a serious condition that causes severe disability and imposes a huge burden on patients, families, and society.^1^, ^2^ The pathophysiological mechanisms of SCI include primary injury, which is the irreversibly mechanical injury, and secondary injuries caused by many factors, such as neuroinflammation,^3^, ^4^ neuronal loss,^5^, ^6^ and oxidative stress injury.^7^ In the pathology of SCI, primary mechanical damage, an irreversible mechanical injury, rapidly leads to disruption of the blood‐spinal cord barrier^8^, ^9^ as well as neuronal loss.^5^, ^6^ Once impaired, neurons, which have a vital role in the central nervous system (CNS), are very difficult to regenerate. Accumulating evidence has suggested that inhibition of excessive inflammatory response^10^ and oxidative stress,^11^ and improving neuronal survival^12^ are the most important strategies in SCI therapy.

Pyroptosis is a newly identified proinflammatory programmed cell death pattern, which is characterized by cell swelling, lysis, the pore formation in the cell membrane, and release of pro‐inflammatory cytokines.^13^, ^14^ Pyroptosis is dependent on caspase‐1 in the classic pathway and caspase‐11 (or caspase‐4/5 in humans) in the non‐canonical pathway.^15^ Nucleotide‐binding oligomerization domain‐like receptor 3 (NLRP3) inflammasome is activated after CNS injury, and the pro‐caspase‐1 changes to active subunits caspase‐1, which can activate Gasdermin D (GSDMD, pore‐forming protein) to generate the N‐terminal fragment of GSDMD (GSDMD‐N), and induce the release of pro‐inflammatory cytokines interleukin (IL)‐1β and IL‐18.^16^, ^17^ Furthermore, pyroptosis contributes to inflammatory response and neuronal loss in SCI^18^ and traumatic brain injury.^19^

Elamipretide (SS‐31, D‐Arg‐dimethylTyr‐Lys‐Phe‐NH2), a novel mitochondria‐targeted peptide, has both aromatic and cationic groups, thereby leading to its ability to target the mitochondrial inner membrane.^20^, ^21^ Owing to its alternating aromaticcationic structure, elamipretide (EPT) can freely cross the blood–brain barrier and cell membranes.^20^, ^22^ Moreover, EPT can eliminate reactive oxygen species (ROS), up‐regulate adenosine triphosphate (ATP), reduce cytochrome c release, prevent mitochondrial swelling, and maintain the mitochondrial membrane potential (MMP) in mitochondria.^23^, ^24^ In addition, EPT has also been reported to protect against traumatic brain injury,^25^ hind limb ischemia–reperfusion injury,^26^ and type 2 diabetes.^27^

Yet, the effect and underlying mechanism of EPT in SCI remains to be elucidated. Hence, the aim of this study was to use in vivo and in vitro models to (1) determine whether EPT provides neuroprotection and attenuates SCI‐induced neuroinflammation and neuronal loss; (2) investigate the signaling mechanisms that mediate the effects induced by EPT following SCI.

## 2 MATERIALS AND METHODS

### 2.1 Animal models and surgical procedure

C57BL/6 female mice, 8 to 10 weeks of age, weighing 20–25 g were obtained from Shanghai SLAC Laboratory Animal Co., Ltd. All the animals were housed in an environment with a temperature of 22 ± 1°C, relative humidity of 50 ± 1%, and a light/dark cycle of 12/12 h. In addition, all animal studies (including the mice euthanasia procedure) were done in compliance with the regulations and guidelines of the Animal Ethics Committee of Hangzhou First People's Hospital's cooperative animal experiment center and conducted according to the Association for Assessment and Accreditation of Laboratory Animal Care International and the Institutional Animal Care and Use Committee guidelines.

For the SCI model, female C57/BL6 mice in EPT and vehicle groups were anesthetized by intraperitoneal injection of 1% sodium pentobarbital (75 mg/kg) to expose the T10–T11 vertebrae laminae. Subsequently, the vascular clamp (30 g force) was placed on both sides of the exposed spinal cord for 1 min based on previous studies.^28^, ^29^ Mice in sham group only underwent laminectomy without crush injury to the spinal cord. After operation, manual urinary bladder emptying (twice a day until recovery) was performed.

Immediately after surgery, EPT (5 mg/kg, synthesized in China by Peptides Company Limited) was intraperitoneally injected once a day in mice for 3 days (totally 3 times) in the EPT group. The same amount of vehicle (saline) was also intraperitoneally injected once a day in mice for 3 days (totally 3 times) in vehicle and sham groups. EPT dosage was chosen according to previous studies.^25^, ^26^ Based on previous study,^30^ histological evaluation and functional assessment were only performed in EPT and vehicle groups.

### 2.2 Functional assessment

Locomotor functions for experimental groups were assessed by two blind observers using the Basso Mouse Scale (BMS).^30^, ^31^ Consensus scores for each mouse based on hindlimb movements were averaged for a maximum of 9 points for the BMS score. The BMS was evaluated at day 1, 3, 7, 14, 21, and 28 post‐injury with nine mice in each group (*n* = 9 mice/ group).

### 2.3 Tissue collection

At 3 or 28 days post‐SCI, mice were euthanized with an overdose of sodium pentobarbital. At 3 days after SCI, spinal cord tissue samples were quickly collected and stored at −80°C for western blot, quantitative real‐time polymerase chain reaction (Q‐PCR), and enzyme‐linked immunosorbent assay (ELISA) (10 mm, centered at the lesion epicenter) and flow cytometry (5 mm, centered at the lesion epicenter). At day 28 after SCI, spinal cord samples (5 mm, centered at the lesion epicenter) were fixed in formaldehyde solution, subsequently dehydrated, embedded, and transversely sliced (thickness, 4 μm) for immunochemistry staining.

### 2.4 Immunostaining

For immunochemistry staining, spinal cord sections (5 mm, centered at the lesion epicenter) were incubated with 0.3% hydrogen peroxide for 10 min, washed in phosphate buffer saline (PBS), and then treated with ethylenediaminetetraacetic acid (EDTA) antigen retrieval solution, added appropriate amount of endogenous peroxidase blockers and incubate at room temperature for 10 min, followed by incubation with anti‐NeuN (1:100, Abcam, catalog #ab177487) at 4°C overnight. After washing in PBS, sections were added appropriate amount of enzyme labeled sheep anti‐mouse IgG polymer (Beijing Zhongshu Jinqiao Biotechnology Co., Ltd., catalog #PV‐6002), incubate at room temperature for 20 min, and then added appropriate amount of freshly prepared chromogenic solution. Sections were visualized with a fluorescence microscope (Olympus Inc.) for immunochemistry staining. To obtain quantitative analysis of neurons in the ventral horn, the number of positive cells from the optical field of the same area in the section at 400um caudal to the injury epicenter of spinal cord were counted to obtain the final data (*n* = 5 mice/group).

For immunofluorescence staining of cells, the slides with climbed cells were washed and fixed with 4% paraformaldehyde for 20 min, and then permeabilized with 0.1% Triton X‐100 at room temperature for 20 min, followed by closed with sealing fluid for 1 h. Then, the slides were washed and incubated with anti‐NeuN (1:100, Novus, catalog # NBP3‐05554‐100) and anti‐GSDMD (1:100, Abcam, catalog # ab219800) or anti‐NeuN (1:100, Absin, catalog # ab145611) and anti‐NLRP3 (1:100, Absin, catalog # abs151715) and at 4°C overnight. Sections were incubated in the secondary antibody at room temperature for 2 h, and then incubated with DAPI (4′,6‐diamidino‐2‐phenylindole) for 10 min. Visualization of slides was performed with a fluorescence microscope (Olympus Inc.).

### 2.5 Primary neuronal culture and injury models

Primary cortical neuronal cultures were prepared and cultured as previously described.^32^ Cerebral cortices collected from embryonic day 14 mouse were minced, dissociated with 0.25% trypsin (Invitrogen), and passed through a cell strainer. Cells were plated on poly‐L‐lysine‐coated dishes at a density of 1 × 10^6^ /mL and maintained in basal neuronal medium (supplemented with 1% L‐glutamine, 2% B27, and 1% penicillin–streptomycin) at 37°C in a humidified incubator (5% CO_2_ and 95% air). The medium was replaced every 2 days and primary cortical neurons were cultured for additional 7 days before use.

To further explain the effects and underlying mechanisms of EPT on pyroptosis, we exposed the primary cultured neurons at oxygen–glucose deprivation (OGD) condition based on previous studies.^18^, ^33^ For OGD, the culture medium was replaced with glucose‐free Dulbecco's Modified Eagle Medium (Gibco), and incubated in a hypoxic chamber (5% CO_2_, 94.98% N_2_ and 0.02% O_2_) at 37°C for 6 h. Neurons were pretreated with EPT (50 μM) for 24 h before OGD based on a previous study.^34^

### 2.6 Quantitative real‐time polymerase chain reaction (Q‐PCR)

Nuclei acid from collected cells, spinal cord samples, and mitochondria samples were obtained by using Trizol agents (Invitrogen). Primer 3 software was applied to design and/or evaluation of a specific pair of primers (Table 1). Complementary DNA (cDNA) was synthesized from 1 μg of total RNA through a TaKaRa reverse transcription (RT) kit and random hexanucleotide primers. The expression levels of genes were detected in a mixture consisting of reversed transcribed cDNAs, SensiMix SYBR and fluorescein, RNase‐free water, and primers. Real‐time reverse transcription PCR was conducted for the quantitative analysis of both mRNA expression of specific genes in the collected cells and spinal cord samples and mitochondrial (mt) DNA in the collected mitochondria samples. Actin level was used for normalization of mRNA expression levels of NLRP3 and ASC. Additionally, the values of mtDNA were normalized to the expression level of 18S rRNA, which was encoded by nuclear DNA.

**TABLE 1:** Real‐time PCR primer sequences.

Gene	Forward primer (5′–3′)	Reverse primer (5′–3′)
NLRP3	ATCAACAGGCGAGACCTCTG	GTCCTCCTGGCATACCATAGA
ASC	GAATGTGCCCAACTTGTTACAC	CAATCTGAACGGAGAGAATCCC
β‐actin	AGAGGGAAATCGTGCGTGAC	CCAAGAAGGAAGGCTGGAAA
Mitochondrial DNA	CACCATTAGCACCCAAAGCT	TGATTTCACGGATGGTG
18S	ATGCGGCGGCGTTATTCC	GCTATCAATCTGTCAATCCTGTCC

### 2.7 Adenosine triphosphate (ATP) assay

Briefly, cells mixed with substrate solution, accelerator, precipitator, chromogenic solution, and terminator follow the step‐by‐step instructions of manufacturer (Nanjing Jiancheng Bioengieering Institute, catalog #A095‐1‐1). The 250uL mixture was set at room temperature for 5 min and measured the absorbance value at 636 nm.

### 2.8 Pro‐inflammatory cytokines and MDA assay

Levels of IL‐1β and IL‐18 in the supernatant of cell culture medium and spinal cord tissue homogenate were detected by ELISA kits of IL‐1β (catalog # BPE30552H) and IL‐18 (catalog #SEA064Mu) following the manufacturer's instructions (Wuhan Youersheng). The levels of MDA in the primary cultured neurons were quantified using commercial kits according to the manufacturer's instructions (Jiancheng, catalog # A003‐4‐1).

### 2.9 Flow cytometry

Spinal cord tissue specimens were collected, cut, and digested with collagenase. Combination of markers, F4/80− (1:100, BioLegend, catalog #103111), CD45+ (1:100, eBioscience, catalog #11–0112‐85), CD11b+ (1:100, eBioscience, catalog #45–5931‐80), and Gr1+ (1:100, eBioscience, catalog #12‐4801‐80) were applied to determine neutrophils. The cytometer (BD Biosciences) was used for performing the measurement of positive cell.

The primary cultured neurons were harvested and stained with SYTOX® Blue dead cell stain (a high‐affinity nucleic acid that penetrates only compromised plasma membranes; Molecular Probes, Eugene, OR, USA). Subsequently, cells were centrifugally rinsed, fixed with 4% paraformaldehyde, and received break of membrane with 0.5% Triton X‐100 and blocking, and then incubated with primary antibodies against GSDMD (1:100, Abcam, catalog # ab219800) overnight at 4°C. Cells were centrifugally rinsed and incubated the corresponding fluorescent secondary antibody, and then were kept away from light at room temperature for 1 h. Cells were examined and analyzed by a flow cytometer (BD Biosciences). Vertical axis is marked for GSDMD positive, and horizontal axis is marked for SYTOX® Blue in the image. Pyroptosis was defined as double positive for GSDMD and SYTOX® Blue. The cells without any dye and antibody are the negative control group. The cells that have been incubated with SYTOX blue alone, the cells that have been incubated with GSDMD and its corresponding fluorescent secondary antibody are the single label group, respectively, so as to determine the position of the cross gate. The double‐positive group of GSDMD/SYTOX® blue is in the upper right quadrant.

For measurement of mitochondrial reactive oxygen species (mt‐ROS), the primary cultured neurons were similarly harvested, handled with MitoSOX kit (Gibco, catalog # M36008) according to the steps in the manual, and detected through a flow cytometer (BD Biosciences). The vertical axis is marked for count and the horizontal axis is marked for MitoSOX in the image. Taking the cells without any dye as the negative control, MitoSOX mitochondrial superoxide dye was used to quantify the fluorescence value of MitoSOX. The stronger the level of mitochondrial superoxide, the stronger the fluorescence value of MitoSOX.

For measurement of MMP level, the primary cultured neurons were handled with JC‐1 kit (Beyotime, catalog #C2006) according to the instructions, and then detected by a flow cytometer (BD Biosciences). The vertical axis is marked for JC‐1 red and the horizontal axis is marked for JC‐1 green in the image. The cells without any dye were used as the negative control. JC‐1 polymer emits red fluorescence, while JC‐1 monomer emits green fluorescence. P1 gate is the cell group of JC‐1 monomer emitting green fluorescence. When the MMP in the cell is depolarized, the form of JC‐1 monomer increases and the green fluorescence becomes stronger.

### 2.10 Cell viability and damage assays

Cell viability was measured using a commercial Cell Counting Kit‐8 (CCK‐8)‐based test kit (Lianke, catalog # 70‐CCK805). The absorbance at 450 nm was measured. Lactate dehydrogenase (LDH) release detection was determined for the measurement of cell injury using a commercially available kit (Jiancheng, catalog # A020‐1‐2). Quantification of LDH concentration was analyzed by measuring the absorbance at 450 nm.

### 2.11 Western blot

The protein sample of cells, as well as spinal cord tissues (*n* = 5 mice/group), were collected and lysed using RIPA lysate buffer (Beyotime) and measured by BCA Protein Assay Kit (Beyotime). The collected protein samples and collected mitochondria samples were separated by sodium dodecyl sulfate‐polyacrylamide gel electrophoresis (SDS‐PAGE) protein sample buffer and heated for 5 min at 100°C and subsequently transferred to polyvinylidene difluoride (PVDF) membranes. The membranes were washed, blocked with non‐fat dry milk, and then incubated with primary antibodies against NLRP3 (1:1000, Cell Signaling Technology, catalog # 15101), ASC (1:1000, Cell Signaling Technology, catalog # 67824), caspase‐1 (1:1000, Abcam, catalog # ab138483), GSDMD (1:1000, Abcam, catalog # ab219800), GSDMD‐N (1:1000, Cell Signaling Technology, catalog #50928 s), IL‐1β (1:1000, Cell Signaling Technology, catalog # 12507), IL‐18 (1:500, Abcam, catalog # ab71495), cytochrome c (1:1000, Abcam, catalog # ab13575), followed by secondary antibody (1:5000, Lianke, catalog # Goat‐anti Mouse GAM007; Goat‐anti Rabbit GAR0072) incubation. The bands were detected using an enhanced chemiluminescence reagent (Perkin Elmer Life Sciences). Finally, these bands were detected and analyzed using MultiGauge image analysis software version 3.0 (Fujifilm Holdings Corporation).

### 2.12 Statistical analyses

All data in the present study were presented as the mean ± error of the mean (SEM). The Kolmogorov–Smirnov test was used to test normality. On the basis of data being normally or non‐normally distributed, parametric or nonparametric tests were performed, respectively. The BMS score was analyzed by two‐way repeated‐measures ANOVA with Bonferroni's post hoc test for multiple comparisons. The two‐tailed Student's *t*‐tests or the nonparametric Mann–Whitney's test was used for the analysis of single comparisons between two different groups and one‐way ANOVA followed by Dunnett's multiple post hoc test was employed for multiple comparisons of more than two groups. *p*‐value < 0.05 was considered statistically significant.

## 3 RESULTS

### 3.1 EPT reduces motor neuronal loss and improves behavioral recovery following SCI

**FIGURE 1:**
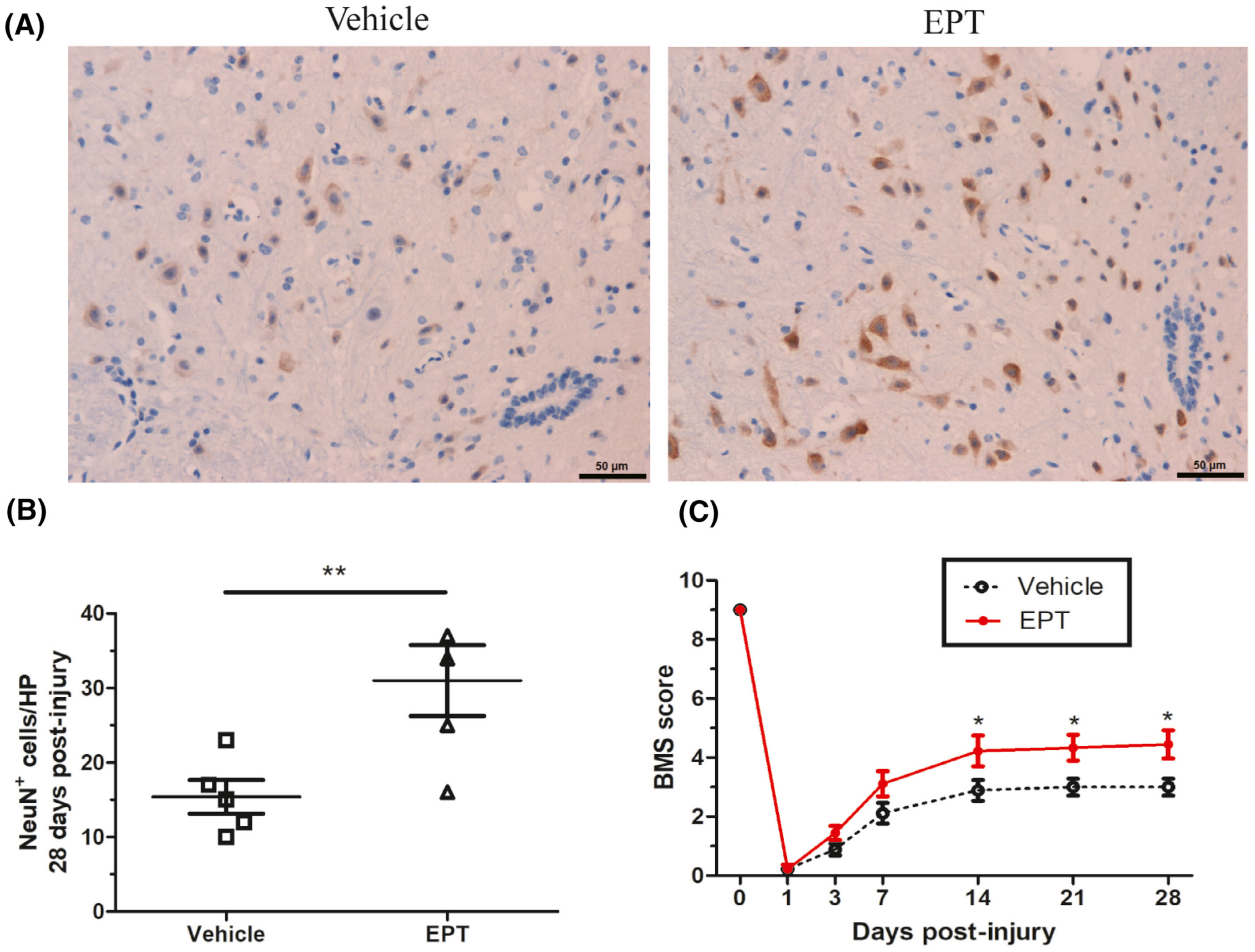
EPT reduces neuronal loss and improves behavioral recovery after SCI in mice. (A) Representative micrographs showing sparing of ventral horn neurons in elamipretide‐treated and vehicle‐treated mice in tissue sections at 400 μm caudal to the injury epicenter 28 days post‐injury, scale bars: 50 μm. (B) Mice treated with elamipretide show significant improvement in locomotor functions assessed by the BMS score, *n* = 9 mice/group. All values are presented as the mean ± SEM. *p < 0.05, **p < 0.01 versus corresponding sham or vehicle group.

First, we estimated the effects of EPT on motor neuronal loss through immunochemistry staining and locomotor recovery with the BMS score. As shown in Figure 1A,B, compared to vehicle animals, treatment with EPT leads to significant increase in NeuN‐positive cells 28 days (Figure 1B; *p* < 0.01) after SCI in EPT‐treated animal.

Furthermore, locomotor assessment at 1, 3, and 7 days post‐injury showed no significant difference in BMS score between EPT and vehicle groups. Remarkably, in the EPT group, EPT treatment led to significantly increased BMS score compared to the vehicle group on days 14, 21, and 28 post‐injury (Figure 1C). Therefore, EPT can promote functional recovery and reduce neuronal loss in mice after SCI.

### 3.2 EPT reduces the number of neutrophils in mice after SCI

**FIGURE 2:**
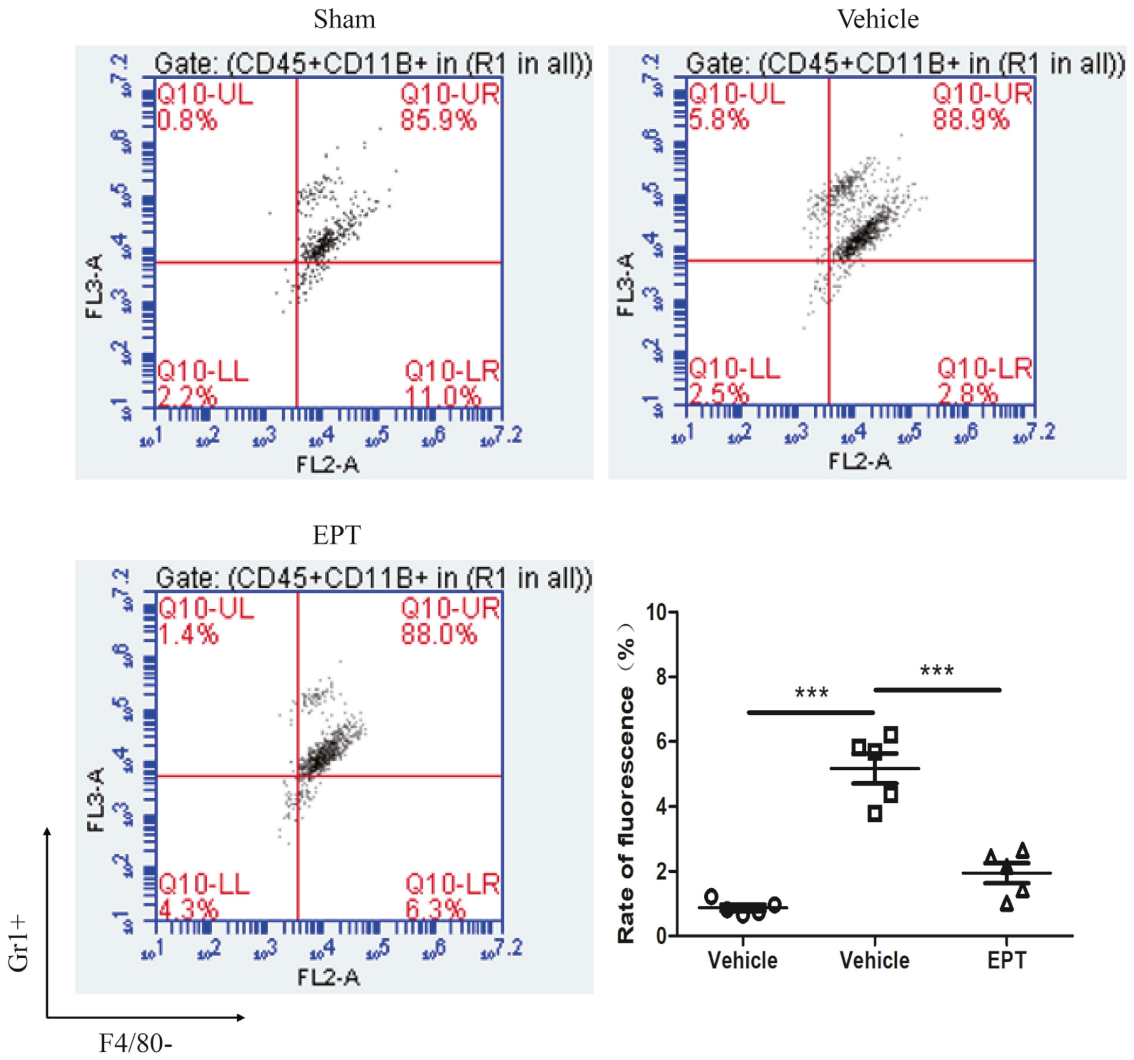
EPT inhibits and reduces the number of neutrophils in mice after SCI. Representative fluorescence‐activated cell sorter (FACS) analysis of the neutrophil counts from the contused spinal cord in mice at 3 days after injury. All values are presented as the mean ± SEM. ***p < 0.001 versus corresponding sham or vehicle group.

To assess whether the resolution of neutrophil inflammation after SCI is linked to EPT treatment, we investigated the neutrophil counts in the injured spinal cord at day 3. SCI induced a significant increase in number of neutrophil in the vehicle group compared with sham group (*p* < 0.001, Figure 2). However, EPT reduced the neutrophil counts from the contused spinal cord in mice (*p* < 0.001, Figure 2).

### 3.3 EPT inhibits pyroptosis and reduces proinflammatory cytokines levels after SCI in mice

To investigate the effects of EPT on pyroptosis in mice, we examined the levels of the pyroptosis‐related proteins GSDMD, GSDMD‐N using western blot analysis in spinal cord samples.

**FIGURE 3:**
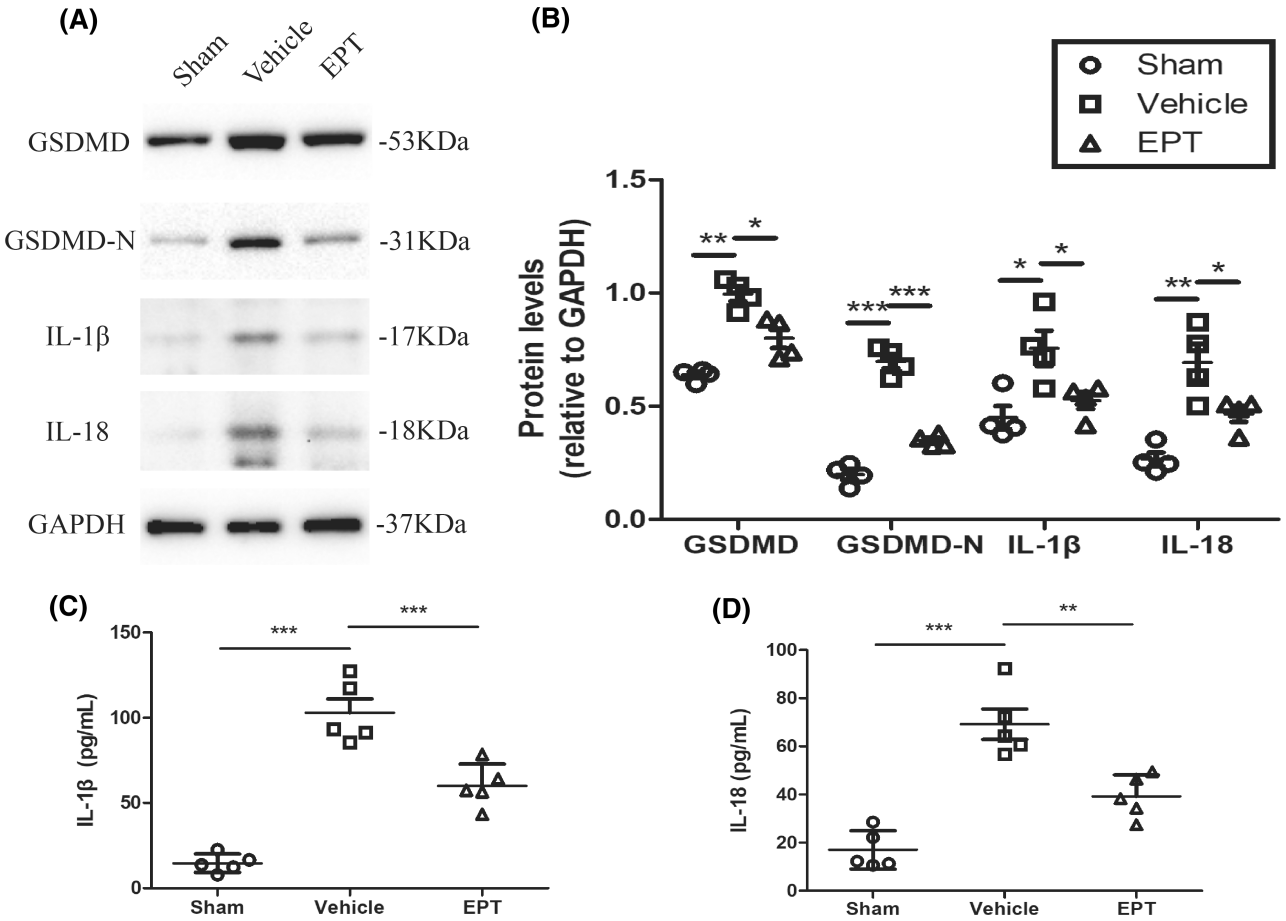
EPT inhibits pyroptosis and controls neuroinflammation after SCI in mice. (A, B) Western blot analysis showing pyroptosis‐related protein GSDMD, GSDMD‐N, and pro‐inflammatory cytokines IL‐1β and IL‐18 in contused spinal cord at 3 days after injury, *n* = 5 mice/ group. (C, D) ELISA analysis showing IL‐1β and IL‐18 in contused spinal cord at 3 days after injury. All values are presented as the mean ± SEM. *p < 0.05, **p < 0.01, and ***p < 0.001 versus corresponding sham or vehicle group.

Protein levels of GSDMD, GSDMD‐N, IL‐1β, and IL‐18 were significantly increased in vehicle group on day 3 post‐injury compared with the sham group, as shown in Figure 3A,B (*p* < 0.01 for GSDMD, *p* < 0.001 for GSDMD‐N, *p* < 0.05 for IL‐1β, *p* < 0.01 for IL‐18). Nevertheless, EPT led to lower levels in EPT group compared to those in the vehicle group (*p* < 0.05 for GSDMD, *p* < 0.001 for GSDMD‐N, *p* < 0.05 for IL‐1β, *p* < 0.05 for IL‐18, Figure 3A,B).

Moreover, the vehicle group showed an increase in the levels of proinflammatory cytokines IL‐1β and IL‐18 detected with ELISA compared to that the sham group (*p* < 0.001 for IL‐1β, *p* < 0.001 for IL‐18, Figure 3C,D), while EPT treatment caused a significant reduction in the protein expression of IL‐1β and IL‐18 compared to that in the vehicle group (*p* < 0.001 for IL‐1β, *p* < 0.01 for IL‐18, Figure 3C,D). These results showed that EPT treatment significantly inhibited pyroptosis and attenuated neuroinflammation following SCI in mice.

### 3.4 EPT inhibits NLRP3 inflammasome activation after SCI in mice

To investigate the potential mechanism of EPT on pyroptosis, we examined the levels of NLRP3 inflammasome‐related proteins NLRP3, ASC, and caspase‐1 by using western blot and NLRP3, ASC using Q‐PCR. Previous studies have demonstrated that the expression of NLRP3 and caspase‐1, which mediates pyroptosis, peaks at 3 days after injury in SCI models.^35^, ^36^

**FIGURE 4:**
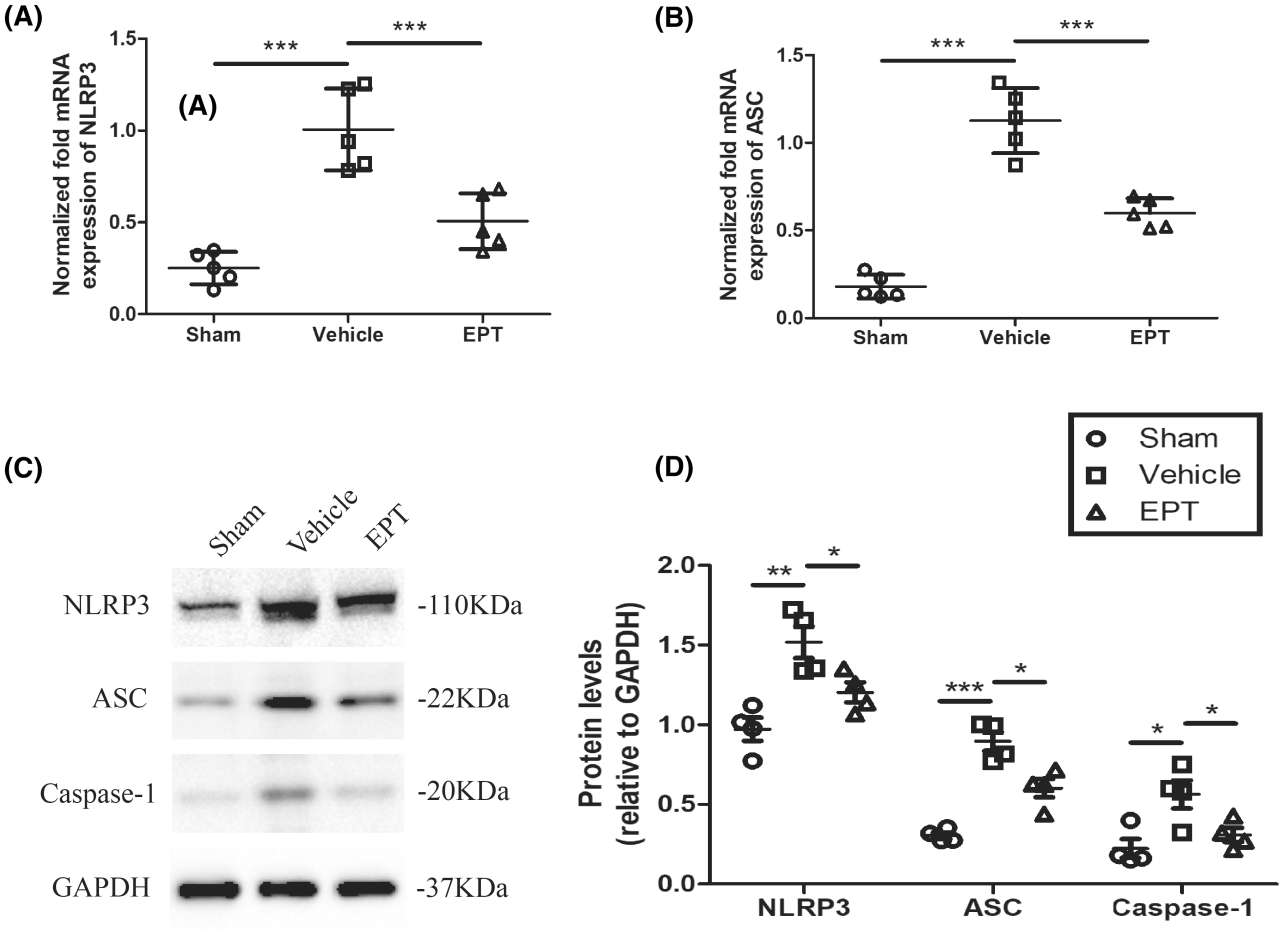
EPT inhibits NLRP3 inflammasome activation after SCI in mice. (A, B) Q‐PCR analysis showing NLRP3 and ASC in contused spinal cord at 3 days after injury. (C, D) Western blot analysis showing NLRP3, ASC, caspase‐1 in contused spinal cord at 3 day after injury. All values are presented as the mean ± SEM. *p < 0.05, **p < 0.01, and ***p < 0.001 versus corresponding sham or vehicle group.

Compared with sham group mice, mice in the vehicle group expressed high levels of NLRP3 and ASC (*p* < 0.001 for NLRP3, *p* < 0.001 for ASC, Figure 4A,B), however, these increases in mRNA expression were suppressed by EPT treatment (*p* < 0.001 for NLRP3, *p* < 0.001 for ASC, Figure 4A,B).

A significant increase in the levels of NLRP3, ASC, and active‐caspase‐1 was observed at 3 days after injury in the vehicle group compared with the sham group (*p* < 0.01 for NLRP3, *p* < 0.001 for ASC, *p* < 0.05 for active‐caspase‐1, Figure 4C,D). Yet, EPT‐treated animals showed a significant reduction of these proteins expression compared with the vehicle group (*p* < 0.05 for NLRP3, *p* < 0.05 for ASC, *p* < 0.05 for active‐caspase‐1, Figure 4C,D). These results support the idea that EPT inhibited NLRP3 inflammasome activation after SCI.

### 3.5 EPT inhibits pyroptosis and attenuates inflammation in the primary cultured neurons

To further confirm the effects of EPT on pyroptosis after SCI, the primary cultured neurons at the condition of OGD were used in vitro based on previous studies.^18^, ^33^ To detect the effects of EPT on pyroptosis in primary cultured neurons, we examined the levels of the pyroptosis‐related proteins GSDMD, GSDMD‐N using western blot analysis, immunofluorescence staining of GSDMD and NeuN, and flow cytometry of double‐positive cells of SYTOX Blue staining and GSDMD.

**FIGURE 5:**
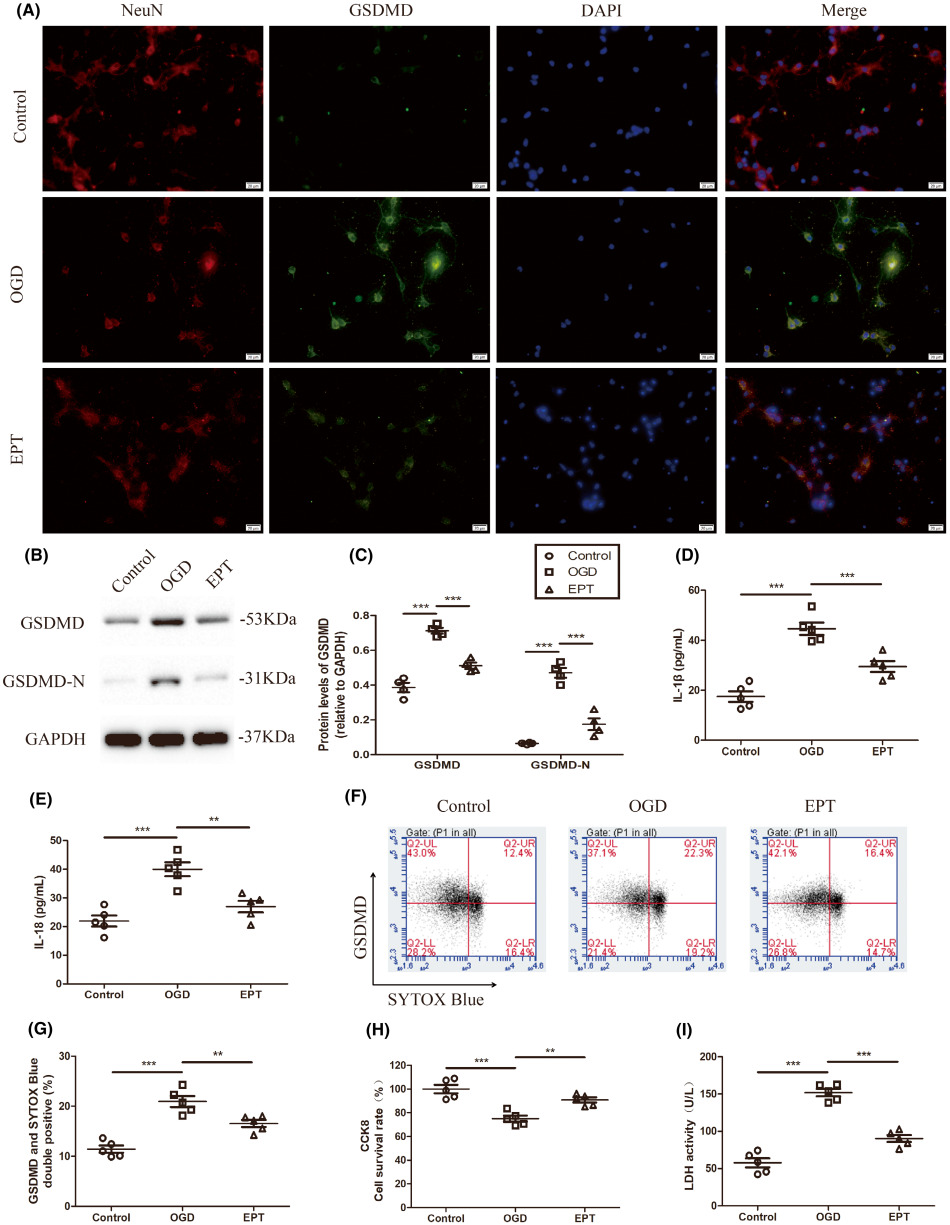
EPT inhibits pyroptosis and attenuates inflammatory response in the primary cultured neurons. (A) Representative micrographs showing immunofluorescence staining of NeuN and GSDMD, scale bars: 20 μm. (B, C) Western blot analysis showing GSDMD and GSDMD‐N levels. (D, E) ELISA analysis of pro‐inflammatory cytokine IL‐1β and IL‐18. (F, G) Representative fluorescence‐activated cell sorter (FACS) analysis dot plots showing the dynamics of double‐positive cells of SYTOX Blue staining and GSDMD. (H) Graph showing the quantitative analysis of cell viability determined by CCK8 assay. (I) Graph showing the quantitative analysis of LDH activity in culture supernatants. All values are presented as the mean ± SEM. **p < 0.01, and ***p < 0.001 versus corresponding control or OGD group.

We performed immunofluorescence staining for both the neuron marker NeuN and the pyroptosis‐associated marker GSDMD in the primary cultured neurons after ODG. The fluorescence intensity of NeuN decreased after ODG, and EPT treatment inhibited the change in EP‐treated cells (Figure 5A). On the contrary, the fluorescence intensity of GSDMD increased after OGD, and the fluorescence was down‐regulated in EP‐treated neurons compared with the OGD group (Figure 5A).

Western blot analysis revealed that OGD led to significant increases in the protein expression of GSDMD and GSDMD‐N, relative to control cells (*p* < 0.001 for GSDMD, *p* < 0.001 for GSDMD‐N, Figure 5B,C). However, EPT treatment significantly downregulated OGD‐mediated increase (*p* < 0.001 for GSDMD, *p* < 0.001 for GSDMD‐N, Figure 5B,C).

Furthermore, ELISA analysis showed that OGD induced the higher levels of IL‐1β (*p* < 0.001, Figure 5D) and IL‐18 (*p* < 0.05, Figure 5E) in the OGD group in the culture supernatant compared to control group, whereas EPT administration mitigated OGD‐induced increase in IL‐1β (*p* < 0.001, Figure 5D) and IL‐18 (*p* < 0.01, Figure 5E) in the EPT group compared to the OGD group.

Moreover, compared with the control group, a significant increase in the double‐positive cells of SYTOX Blue staining and GSDMD was observed in the OGD group (*p* < 0.001, Figure 5F,G). However, the OGD‐mediated increase in double‐positive cells was significantly decreased by treatment with EPT in the primary cultured neurons (*p* < 0.01, Figure 5F,G).

Next, CCK‐8 and LDH assays were performed to investigate the effects of EPT on cell viability and injury, respectively. We found that OGD caused a decline in the value of CCK‐8 (*p* < 0.001, Figure 5H) and an increase in LDH release (*p* < 0.001, Figure 5I) in the culture supernatant in the OGD group compared to control group. Nevertheless, EPT significantly controlled the alterations induced by OGD (*p* < 0.01 for CCK‐8, *p* < 0.001 for LDH, Figure 5H,I) in the EPT group compared to OGD group.

### 3.6 EPT inhibits NLRP3 inflammasome activation in the primary cultured neurons

To investigate the potential mechanism of EPT on pyroptosis, we examined the levels of NLRP3 inflammasome‐related protein by using immunofluorescence staining, western blot analysis, and Q‐PCR in the primary cultured neurons.

**FIGURE 6:**
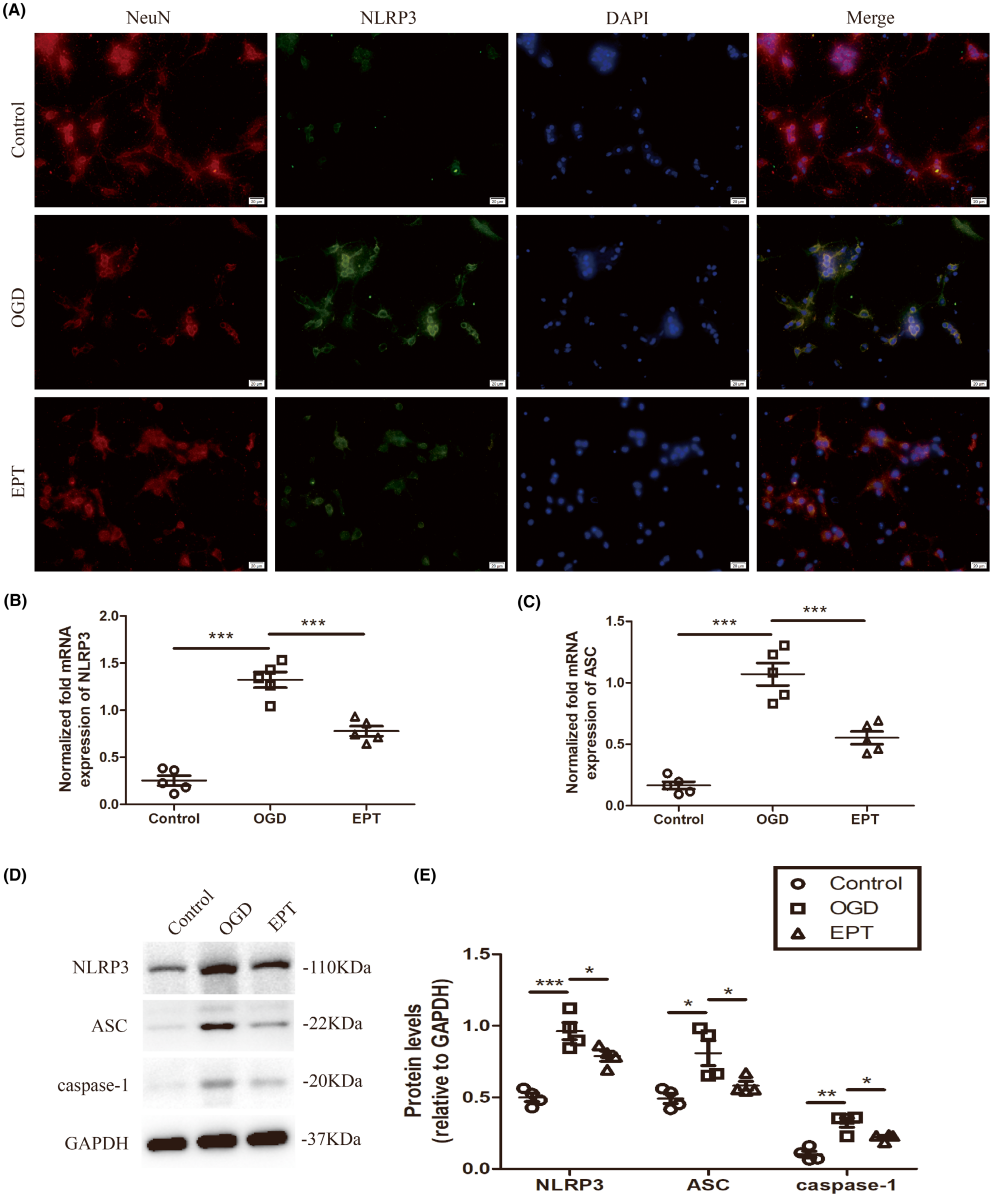
EPT inhibits NLRP3 inflammasome activation in the primary cultured neurons. (A) Representative micrographs showing immunofluorescence staining of NeuN and NLRP3, scale bars: 20 μm. (B, C) Q‐PCR analysis showing NLRP3 and ASC mRNA expression. (D, E) Western blot analysis showing NLRP3, ASC, caspase‐1 levels. All values are presented as the mean ± SEM. *p < 0.05, **p < 0.01, and ***p < 0.001 versus corresponding control or OGD group.

Immunofluorescence staining analysis revealed that the OGD group showed an increase in the fluorescence intensity of NLRP3 compared to that of the control group, while EPT treatment caused a significant reduction in the fluorescence intensity of NLRP3 compared to that in the OGD group (Figure 6A).

The mRNA expression of NLRP3 and ASC in the OGD group was significantly higher than those in the control group (*p* < 0.001 for NLRP3, *p* < 0.001 for ASC, Figure 6B,C). After EPT application, the mRNA expression of NLRP3 and ASC was significantly lower than those in the OGD group (*p* < 0.001 for NLRP3, *p* < 0.001 for ASC, Figure 6B,C).

Western blot analysis revealed that OGD led to significant increases in the protein levels of NLRP3, ASC, and active‐caspase‐1 in the OGD group relative to control group (*p* < 0.001 for NLRP3, *p* < 0.05 for ASC, *p* < 0.01 for caspase‐1, Figure 6D,E). However, EPT treatment significantly downregulated OGD‐mediated increase in these proteins in the EPT group compared to OGD group (*p* < 0.05 for NLRP3, *p* < 0.05 for ASC, *p* < 0.05 for caspase‐1, Figure 6D,E).

Together, our results suggest that EPT inhibits NLRP3 inflammasome activation after OGD in the primary cultured neurons.

### 3.7 EPT alleviates mitochondrial dysfunction in the primary cultured neurons

To further investigate the mechanism of EPT on NLRP3 inflammasome activation, mitochondrial function was assessed in the primary cultured neurons.

**FIGURE 7:**
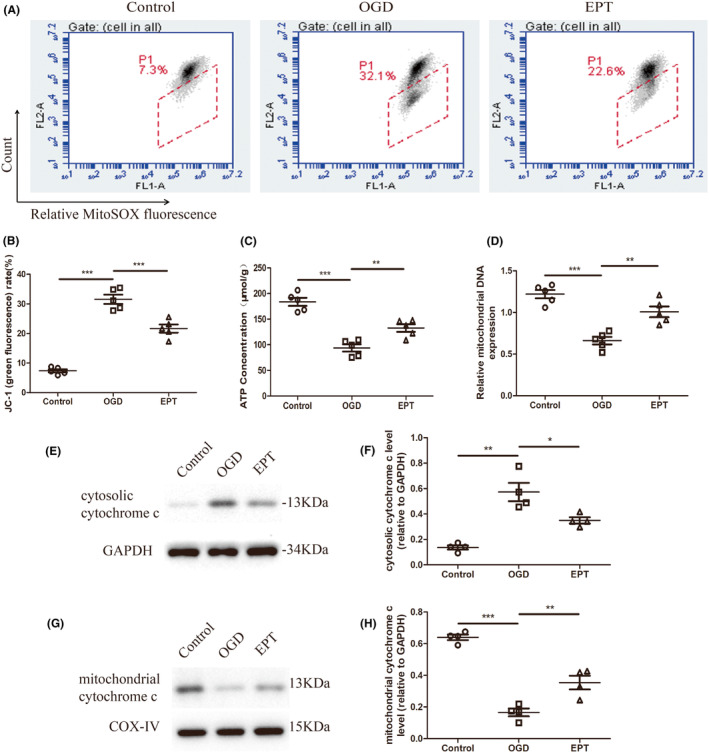
EPT alleviates mitochondrial dysfunction in the primary cultured neurons. (A, B) Flow cytometry analysis showing the JC‐1 (green fluorescence) level. (C) Graph showing the quantitative analysis of ATP level. (D) Graph showing the analysis of mitochondrial DNA expression. (E, F) Western blot analysis showing cytosolic cytochrome c level; (G, H) Western blot analysis showing mitochondrial cytochrome c. All values are presented as the mean ± SEM. *p < 0.05, **p < 0.01 and ***p < 0.001 versus corresponding control or OGD group.

As shown in Figure 7A,B, obvious abnormality in MMP, as indicated by a significant increase in JC‐1 level (green fluorescence), was observed in the OGD group (*p* < 0.001). However, EPT treatment significantly down‐regulated JC‐1 level (green fluorescence) compared to that in the OGD group (*p* < 0.001, Figure 7A,B). After the injury, a significant decrease in the ATP concentration and mitochondrial DNA expression was observed in the OGD group compared with control cells (*p* < 0.001 for ATP, *p* < 0.001 for mitochondrial DNA, Figure 7C,D). Nevertheless, the decrease due to OGD was markedly inhibited by EPT administration (*p* < 0.01 for ATP, *p* < 0.01 for mitochondrial DNA, Figure 7C,D).

Western blot analysis revealed that OGD led to a significant higher level of cytosolic cytochrome c (*p* < 0.01, Figure 7E,F) and lower level of mitochondrial cytochrome c (*p* < 0.001, Figure 7E,F) in the OGD group relative to control group. However, EPT treatment resulted in reversion of cytosolic cytochrome c (*p* < 0.05, Figure 7G,H) and mitochondrial cytochrome c (*p* < 0.01, Figure 7G,H) levels in the EPT group compared to OGD group. Together, these results indicate that EPT attenuates mitochondrial dysfunction in the primary cultured neurons.

### 3.8 EPT alleviates mt‐ROS level in the primary cultured neurons

**FIGURE 8:**
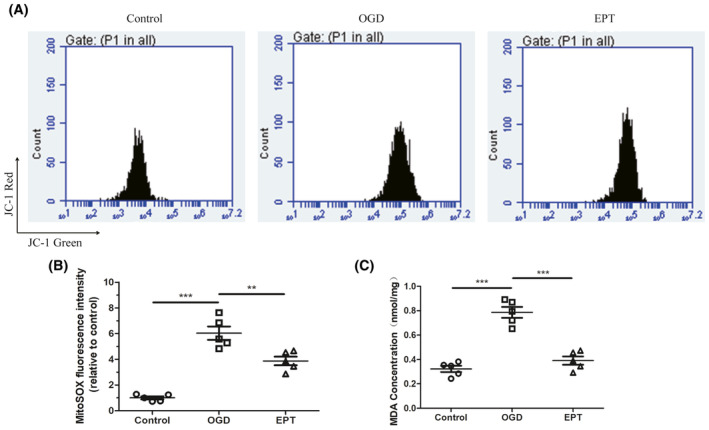
EPT alleviates mt‐ROS level in the primary cultured neurons. (A, B) Flow cytometry analysis mt‐ROS level. (C) Graph showing the quantitative analysis of malondialdehyde (MDA) level. All values are presented as the mean ± SEM. **p < 0.01,***p < 0.001 versus corresponding control or OGD group.

OGD induced a significant increase in mt‐ROS level and MDA concentration compared with control cells (*p* < 0.001 for mt‐ROS, Figure 8A,B; *p* < 0.001 for MDA, Figure 8C). However, the ODG‐mediated rise of mt‐ROS and MDA level was significantly dampened by EPT (*p* < 0.001 for mt‐ROS, Figure 8A,B; *p* < 0.001 for MDA, Figure 8C).

## 4 DISCUSSION

In this study, EPT attenuated mitochondrial dysfunction and mt‐ROS level, prevented NLRP3 inflammasome activation, inhibited pyroptosis, and controlled neuroinflammation after SCI. Importantly, our data indicated that EPT reduced neuronal loss and promoted locomotor recovery after SCI. Thus, these results suggest that EPT has protective effects against SCI.

Pro‐inflammatory cytokines IL‐1β and IL‐18 are critical factors of released intracellular contents after pyroptosis.^37^ IL‐1β has detrimental effects on lesion development (in terms of glial activation and size), but also on the plasticity of axons after SCI.^38^ Moreover, IL‐1β can inhibit the functional recovery of neural stem cell transplant therapy for SCI treatment in rats.^39^ The expression of IL‐1β can deteriorate the prognosis of SCI.^40^ Downregulation of IL‐1β level after traumatic SCI may have protective effects in reducing secondary impairments and improving the outcomes.^41^ Furthermore, the expression of IL‐18 is closely related to the severity of SCI.^42^ Our results revealed that EPT led to a reduction in the expressions of IL‐1β and IL‐18 after SCI, thus suggesting a potent action of EPT in the inhibition of neuroinflammation.

To further investigate the mechanism of EPT on the neuroinflammation and neuronal loss, we analyzed pyroptosis in vivo and in vitro. Pyroptosis is a novel recognized pro‐inflammatory cell death pattern. The canonical pathway of pyroptosis occurrence is mediated by caspase‐1 activation, leading to lytic cell death through the effector protein GSDMD.^43^ Furthermore, pyroptosis is accompanied by the release of pro‐inflammatory cytokines IL‐1β and IL‐18.^44^ Moreover, pyroptosis is closely associated with neuronal loss^45^ and is an key pathway for neuronal death in SCI.^18^ 27‐Hydroxycholesterol is implicated in the pathogenesis of neuronal death by inducing pyroptosis.^45^ Valproic acid attenuates neuronal impairment caused by ischemic/reperfusion injury through anti‐pyroptotic effects.^46^ Our results revealed that SCI caused pyroptosis in vivo and in vitro. However, EPT reduced pyroptosis after SCI. Moreover, the administration of EPT decreased the neuronal loss and improved neuronal survival. In sum, this is the first study to verify that EPT can inhibit pyroptosis and reduce neuronal loss after SCI.

To investigate the mechanisms underlying the effect of EPT on pyroptosis, the protein expressions of NLRP3, ASC, and caspase‐1 in the spinal cord and primary cultured neurons were assessed by western blot. NLRP3 is currently the most widely studied member of the NLR family.^47^ Earlier studies have shown that the NLRP3 inflammasome was activated after SCI.^35^ Moreover, the NLRP3 inflammasome, a cellular polyprotein complex, can serve as a platform for caspase‐1 activation and the maturation of IL‐1β and IL‐18.^48^ Recent studies have demonstrated that NLRP3 inflammasome can induce pyroptosis.^49^ Pyroptosis induced by radiation was mediated by NLRP3 inflammasome activation in bone marrow‐derived macrophages.^50^ Furthermore, a previous report showed that the limitation of NLRP3 inflammasome activation could control pyroptosis and inflammatory responses.^51^ Here, we reported that EPT significantly inhibits NLRP3 inflammasome activation in vivo and in vitro. Our results further suggested that the mechanisms of suppression of pyroptosis by EPT after SCI presumably depend on the attenuation of the NLRP3 inflammasome activation.

To further explore the mechanism of EPT on NLRP3 inflammasome activation, mitochondrial function, and mt‐ROS level were assessed in the primary cultured neurons. EPT, a novel mitochondrial‐targeted peptide, can concentrate >1000‐fold in the mitochondrial inner membrane.^22^ Moreover, EPT has a dimethyltyrosine residue that allows it to scavenge oxyradicals and suppress linoleic acid and low‐density lipoprotein oxidation.^20^ EPT has been reported to have excellent neuroprotective effects in different disease models of Huntington's disease^52^ and sepsis‐associated encephalopathy^53^ that are associated with inflammation and oxidative stress processes. Moreover, mitochondria are the major source of ROS.^54^ More importantly, overabundance of mt‐ROS can activate NLRP3 inflammasome activation and subsequently pyroptosis pathways.^55^, ^56^, ^57^ Here, our data revealed that EPT attenuated SCI‐induced overabundance of mt‐ROS and mitochondrial dysfunction in the primary cultured neurons.

## 5 CONCLUSION

In summary, this is the first report demonstrating that EPT alleviates pyroptosis and neuroinflammation after SCI. Moreover, EPT reduces neuronal loss and improves locomotor recovery of mice after SCI. Together, our data indicated that EPT may provide a novel approach to promote functional recovery after SCI.

## AUTHOR CONTRIBUTIONS

WJ and JW contributed to the conception of the study. WJ, GD, and FH conducted the experiments and analyzed the data. WJ and JW prepared the manuscript. All authors contributed to critical revision of the manuscript and approved the submitted version.

## CONFLICT OF INTEREST STATEMENT

The authors declare no conflicts of interest.

## Data Availability

The data that support the findings of this study are available from the corresponding author upon reasonable request.

## References

[1] Fu H , Hu D , Zhang L , Shen X , Tang P . Efficacy of oligodendrocyte progenitor cell transplantation in rat models with traumatic thoracic spinal cord injury: a systematic review and meta‐analysis. J Neurotrauma. 2018;35(21):2507‐2518. 29759026 10.1089/neu.2017.5606

[2] Kijima K , Kubota K , Hara M , et al. The acute phase serum zinc concentration is a reliable biomarker for predicting the functional outcome after spinal cord injury. EBioMedicine. 2019;41:659‐669. 30902739 10.1016/j.ebiom.2019.03.003 PMC6444130

[3] Lago N , Pannunzio B , Amo‐Aparicio J , Lopez‐Vales R , Peluffo H . CD200 modulates spinal cord injury neuroinflammation and outcome through CD200R1. Brain Behav Immun. 2018;73:416‐426. 29870752 10.1016/j.bbi.2018.06.002

[4] Tang R , Botchway BOA , Meng Y , et al. The inhibition of inflammatory signaling pathway by secretory leukocyte protease inhibitor can improve spinal cord injury. Cell Mol Neurobiol. 2020;40(7):1067‐1073. 31993863 10.1007/s10571-020-00799-1 PMC11448923

[5] Ahuja CS , Wilson JR , Nori S , et al. Traumatic spinal cord injury. Nat Rev Dis Primers. 2017;3:17018. 28447605 10.1038/nrdp.2017.18

[6] Yoshizaki S , Kijima K , Hara M , et al. Tranexamic acid reduces heme cytotoxicity via the TLR4/TNF axis and ameliorates functional recovery after spinal cord injury. J Neuroinflammation. 2019;16(1):160. 31358003 10.1186/s12974-019-1536-y PMC6661785

[7] Cheng M , Wu X , Wang F , Tan B , Hu J . Electro‐acupuncture inhibits p66Shc‐mediated oxidative stress to facilitate functional recovery after spinal cord injury. J Mol Neurosci. 2020;70(12):2031‐2040. 32488847 10.1007/s12031-020-01609-5

[8] Zhou C , Hu S , Botchway BOA , Zhang Y , Liu X . Valproic acid: a potential therapeutic for spinal cord injury. Cell Mol Neurobiol. 2021;41(7):1441‐1452. 32725456 10.1007/s10571-020-00929-9 PMC11448682

[9] Kumar H , Choi H , Jo MJ , et al. Neutrophil elastase inhibition effectively rescued angiopoietin‐1 decrease and inhibits glial scar after spinal cord injury. Acta Neuropathol Commun. 2018;6(1):73. 30086801 10.1186/s40478-018-0576-3 PMC6080383

[10] Zhang Y , Zhou Y , Chen S , et al. Macrophage migration inhibitory factor facilitates prostaglandin E2 production of astrocytes to tune inflammatory milieu following spinal cord injury. J Neuroinflammation. 2019;16(1):85. 30981278 10.1186/s12974-019-1468-6 PMC6461812

[11] Rios C , Santander I , Mendez‐Armenta M , et al. Metallothionein‐I + II reduces oxidative damage and apoptosis after traumatic spinal cord injury in rats. Oxid Med Cell Longev. 2018;2018:3265918. 30524652 10.1155/2018/3265918 PMC6247576

[12] Wang PF , Xu DY , Zhang Y , et al. Deletion of mammalian sterile 20‐like kinase 1 attenuates neuronal loss and improves locomotor function in a mouse model of spinal cord trauma. Mol Cell Biochem. 2017;431(1–2):11‐20. 28210902 10.1007/s11010-017-2969-1

[13] Wu J , Lin S , Wan B , Velani B , Zhu Y . Pyroptosis in liver disease: new insights into disease mechanisms. Aging Dis. 2019;10(5):1094‐1108. 31595205 10.14336/AD.2019.0116 PMC6764727

[14] Liu X , Zhang Z , Ruan J , et al. Inflammasome‐activated gasdermin D causes pyroptosis by forming membrane pores. Nature. 2016;535(7610):153‐158. 27383986 10.1038/nature18629 PMC5539988

[15] Gong W , Shi Y , Ren J . Research progresses of molecular mechanism of pyroptosis and its related diseases. Immunobiology. 2020;225(2):151884. 31822435 10.1016/j.imbio.2019.11.019

[16] Wang S , Yuan YH , Chen NH , Wang HB . The mechanisms of NLRP3 inflammasome/pyroptosis activation and their role in Parkinson's disease. Int Immunopharmacol. 2019;67:458‐464. 30594776 10.1016/j.intimp.2018.12.019

[17] McKenzie BA , Mamik MK , Saito LB , et al. Caspase‐1 inhibition prevents glial inflammasome activation and pyroptosis in models of multiple sclerosis. Proc Natl Acad sci U S A. 2018;115(26):E6065‐E6074. 29895691 10.1073/pnas.1722041115 PMC6042136

[18] Zheng G , Zhan Y , Wang H , et al. Carbon monoxide releasing molecule‐3 alleviates neuron death after spinal cord injury via inflammasome regulation. EBioMedicine. 2019;40:643‐654. 30612943 10.1016/j.ebiom.2018.12.059 PMC6412161

[19] Liu W , Chen Y , Meng J , et al. Ablation of caspase‐1 protects against TBI‐induced pyroptosis in vitro and in vivo. J Neuroinflammation. 2018;15(1):48. 29458437 10.1186/s12974-018-1083-y PMC5817788

[20] Zhao W , Xu Z , Cao J , et al. Elamipretide (SS‐31) improves mitochondrial dysfunction, synaptic and memory impairment induced by lipopolysaccharide in mice. J Neuroinflammation. 2019;16(1):230. 31747905 10.1186/s12974-019-1627-9 PMC6865061

[21] Tang H , Fang C , Xue S , et al. Protective effects of SS‐31 against SDHB suppression‐mitochondrial dysfunction‐EndMT axis‐modulated CBT sclerosis and progression. Am J Transl Res. 2020;12(11):7603‐7619. 33312392 PMC7724343

[22] Zuo Y , Yin L , Cheng X , et al. Elamipretide attenuates Pyroptosis and perioperative neurocognitive disorders in aged mice. Front Cell Neurosci. 2020;14:251. 32903868 10.3389/fncel.2020.00251 PMC7439217

[23] Yang SK , Han YC , He JR , et al. Mitochondria targeted peptide SS‐31 prevent on cisplatin‐induced acute kidney injury via regulating mitochondrial ROS‐NLRP3 pathway. Biomed Pharmacother. 2020;130:110521. 32717631 10.1016/j.biopha.2020.110521

[24] Machiraju P , Wang X , Sabouny R , et al. SS‐31 peptide reverses the mitochondrial fragmentation present in fibroblasts from patients with DCMA, a mitochondrial cardiomyopathy. Front Cardiovasc Med. 2019;6:167. 31803760 10.3389/fcvm.2019.00167 PMC6873783

[25] Zhu Y , Wang H , Fang J , et al. SS‐31 provides neuroprotection by reversing mitochondrial dysfunction after traumatic brain injury. Oxid Med Cell Longev. 2018;2018:4783602. 30224944 10.1155/2018/4783602 PMC6129854

[26] Cai J , Jiang Y , Zhang M , et al. Protective effects of mitochondrion‐targeted peptide SS‐31 against hind limb ischemia‐reperfusion injury. J Physiol Biochem. 2018;74(2):335‐343. 29589186 10.1007/s13105-018-0617-1

[27] Escribano‐Lopez I , de Maranon AM , Iannantuoni F , et al. The mitochondrial antioxidant SS‐31 modulates oxidative stress, endoplasmic reticulum stress, and autophagy in type 2 diabetes. J Clin Med. 2019;8(9):1322. 31466264 10.3390/jcm8091322 PMC6780723

[28] Paterniti I , Impellizzeri D , Di Paola R , et al. Docosahexaenoic acid attenuates the early inflammatory response following spinal cord injury in mice: in‐vivo and in‐vitro studies. J Neuroinflammation. 2014;11:6. 24405628 10.1186/1742-2094-11-6 PMC3895696

[29] Zhang D , Xuan J , Zheng BB , et al. Metformin improves functional recovery after spinal cord injury via autophagy flux stimulation. Mol Neurobiol. 2017;54(5):3327‐3341. 27167128 10.1007/s12035-016-9895-1

[30] Francos‐Quijorna I , Santos‐Nogueira E , Gronert K , et al. Maresin 1 promotes inflammatory resolution, neuroprotection, and functional neurological recovery after spinal cord injury. J Neurosci. 2017;37(48):11731‐11743. 29109234 10.1523/JNEUROSCI.1395-17.2017 PMC5707767

[31] Basso DM , Fisher LC , Anderson AJ , Jakeman LB , McTigue DM , Popovich PG . Basso mouse scale for locomotion detects differences in recovery after spinal cord injury in five common mouse strains. J Neurotrauma. 2006;23(5):635‐659. 16689667 10.1089/neu.2006.23.635

[32] Slaets H , Nelissen S , Janssens K , et al. Oncostatin M reduces lesion size and promotes functional recovery and neurite outgrowth after spinal cord injury. Mol Neurobiol. 2014;50(3):1142‐1151. 24996996 10.1007/s12035-014-8795-5

[33] Yu D , Li M , Nie P , Ni B , Zhang Z , Zhou Y . Bcl‐2/E1B‐19KD‐interacting protein 3/light chain 3 interaction induces mitophagy in spinal cord injury in rats both in vivo and in vitro. J Neurotrauma. 2018;35(18):2183‐2194. 29566574 10.1089/neu.2017.5280

[34] Wu J , Yan Z , Schwartz DE , Yu J , Malik AB , Hu G . Activation of NLRP3 inflammasome in alveolar macrophages contributes to mechanical stretch‐induced lung inflammation and injury. J Immunol. 2013;190(7):3590‐3599. 23436933 10.4049/jimmunol.1200860 PMC3608749

[35] Jiang W , Li M , He F , Zhou S , Zhu L . Targeting the NLRP3 inflammasome to attenuate spinal cord injury in mice. J Neuroinflammation. 2017;14(1):207. 29070054 10.1186/s12974-017-0980-9 PMC5657095

[36] Zendedel A , Johann S , Mehrabi S , et al. Activation and regulation of NLRP3 inflammasome by intrathecal application of SDF‐1a in a spinal cord injury model. Mol Neurobiol. 2016;53(5):3063‐3075. 25972240 10.1007/s12035-015-9203-5

[37] Lu F , Lan Z , Xin Z , et al. Emerging insights into molecular mechanisms underlying pyroptosis and functions of inflammasomes in diseases. J Cell Physiol. 2020;235(4):3207‐3221. 31621910 10.1002/jcp.29268 PMC13482838

[38] Boato F , Rosenberger K , Nelissen S , et al. Absence of IL‐1beta positively affects neurological outcome, lesion development and axonal plasticity after spinal cord injury. J Neuroinflammation. 2013;10:6. 23317037 10.1186/1742-2094-10-6 PMC3585738

[39] Zhou W , Yuan T , Gao Y , et al. IL‐1beta‐induces NF‐kappaB and upregulates microRNA‐372 to inhibit spinal cord injury recovery. J Neurophysiol. 2017;117(6):2282‐2291. 28298306 10.1152/jn.00936.2016 PMC5461663

[40] Li T , Li YT , Song DY . The expression of IL‐1beta can deteriorate the prognosis of nervous system after spinal cord injury. Int J Neurosci. 2018;128(8):778‐782. 29308940 10.1080/00207454.2018.1424154

[41] Lin WP , Lin JH , Cai B , et al. Effect of adenovirus‐mediated RNA interference of IL‐1beta expression on spinal cord injury in rats. Spinal Cord. 2016;54(10):778‐784. 26902461 10.1038/sc.2016.20

[42] Lin XL , Zhu J , Wang LM , Yan F , Sha WP , Yang HL . MiR‐92b‐5p inhibitor suppresses IL‐18 mediated inflammatory amplification after spinal cord injury via IL‐18BP up‐regulation. Eur Rev Med Pharmacol sci. 2019;23(5):1891‐1898. 30915731 10.26355/eurrev_201903_17226

[43] Shi J , Zhao Y , Wang K , et al. Cleavage of GSDMD by inflammatory caspases determines pyroptotic cell death. Nature. 2015;526(7575):660‐665. 26375003 10.1038/nature15514

[44] McKenzie BA , Dixit VM , Power C . Fiery cell death: pyroptosis in the central nervous system. Trends Neurosci. 2020;43(1):55‐73. 31843293 10.1016/j.tins.2019.11.005

[45] Chen S , Zhou C , Yu H , et al. 27‐hydroxycholesterol contributes to lysosomal membrane permeabilization‐mediated Pyroptosis in Co‐cultured SH‐SY5Y cells and C6 cells. Front Mol Neurosci. 2019;12:14. 30881285 10.3389/fnmol.2019.00014 PMC6405519

[46] Zhu S , Zhang Z , Jia LQ , et al. Valproic acid attenuates global cerebral ischemia/reperfusion injury in gerbils via anti‐pyroptosis pathways. Neurochem Int. 2019;124:141‐151. 30611759 10.1016/j.neuint.2019.01.003

[47] Jin X , Liu MY , Zhang DF , et al. Baicalin mitigates cognitive impairment and protects neurons from microglia‐mediated neuroinflammation via suppressing NLRP3 inflammasomes and TLR4/NF‐kappaB signaling pathway. CNS Neurosci Ther. 2019;25(5):575‐590. 30676698 10.1111/cns.13086 PMC6488900

[48] Lin JQ , Tian H , Zhao XG , et al. Zinc provides neuroprotection by regulating NLRP3 inflammasome through autophagy and ubiquitination in a spinal contusion injury model. CNS Neurosci Ther. 2021;27(4):413‐425. 33034415 10.1111/cns.13460 PMC7941232

[49] Yang Z , Liang C , Wang T , et al. NLRP3 inflammasome activation promotes the development of allergic rhinitis via epithelium pyroptosis. Biochem Biophys Res Commun. 2020;522(1):61‐67. 31740004 10.1016/j.bbrc.2019.11.031

[50] Liu YG , Chen JK , Zhang ZT , et al. NLRP3 inflammasome activation mediates radiation‐induced pyroptosis in bone marrow‐derived macrophages. Cell Death Dis. 2017;8(2):e2579. 28151471 10.1038/cddis.2016.460 PMC5386456

[51] Sun W , Lu H , Lyu L , et al. Gastrodin ameliorates microvascular reperfusion injury‐induced pyroptosis by regulating the NLRP3/caspase‐1 pathway. J Physiol Biochem. 2019;75(4):531‐547. 31440987 10.1007/s13105-019-00702-7

[52] Yin X , Manczak M , Reddy PH . Mitochondria‐targeted molecules MitoQ and SS31 reduce mutant huntingtin‐induced mitochondrial toxicity and synaptic damage in Huntington's disease. Hum Mol Genet. 2016;25(9):1739‐1753. 26908605 10.1093/hmg/ddw045 PMC4986329

[53] Wu J , Zhang M , Hao S , et al. Mitochondria‐targeted peptide reverses mitochondrial dysfunction and cognitive deficits in sepsis‐associated encephalopathy. Mol Neurobiol. 2015;52(1):783‐791. 25288156 10.1007/s12035-014-8918-z

[54] Chiao YA , Zhang H , Sweetwyne M , et al. Late‐life restoration of mitochondrial function reverses cardiac dysfunction in old mice. Elife. 2020;9:e55513. 32648542 10.7554/eLife.55513 PMC7377906

[55] Zheng Z , Bian Y , Zhang Y , Ren G , Li G . Metformin activates AMPK/SIRT1/NF‐kappaB pathway and induces mitochondrial dysfunction to drive caspase3/GSDME‐mediated cancer cell pyroptosis. Cell Cycle. 2020;19(10):1089‐1104. 32286137 10.1080/15384101.2020.1743911 PMC7217368

[56] Jang Y , Lee AY , Jeong SH , et al. Chlorpyrifos induces NLRP3 inflammasome and pyroptosis/apoptosis via mitochondrial oxidative stress in human keratinocyte HaCaT cells. Toxicology. 2015;338:37‐46. 26435000 10.1016/j.tox.2015.09.006

[57] Mishra SR , Mahapatra KK , Behera BP , et al. Mitochondrial dysfunction as a driver of NLRP3 inflammasome activation and its modulation through mitophagy for potential therapeutics. Int J Biochem Cell Biol. 2021;136:106013. 34022434 10.1016/j.biocel.2021.106013

